# Single-cell analysis of nasal epithelial cell development in domestic pigs

**DOI:** 10.1186/s13567-024-01403-w

**Published:** 2024-10-30

**Authors:** Wenqian Wang, Ruiling Liu, Qiu Zhong, Yunlei Cao, Jiaxin Qi, Yuchen Li, Qian Yang

**Affiliations:** Present Address: MOE Joint International Research Laboratory of Animal Health and Food Safety, College of Veterinary Medicine, Nanjing Agricultural University, Nanjing, Jiangsu China

**Keywords:** Nasal epithelial cells, ScRNA-seq, cross-species comparison, respiratory virus receptors

## Abstract

The nasal mucosa forms a critical barrier against the invasion of respiratory pathogens. Composed of a heterogeneous assortment of cell types, the nasal mucosa relies on the unique characteristics and complex intercellular dynamics of these cells to maintain their structural integrity and functional efficacy. In this study, single-cell RNA sequencing (scRNA-seq) of porcine nasal mucosa was performed, and nineteen distinct nasal cell types, including nine epithelial cell types, five stromal cell types, and five immune cell types, were identified. The distribution patterns of three representative types of epithelial cells (basal cells, goblet cells, and ciliated cells) were subsequently detected by immunofluorescence. We conducted a comparative analysis of these data with published human single-cell data, revealing consistent differentiation trajectories among porcine and human nasal epithelial cells. Specifically, basal cells serve as the initial stage in the differentiation process of nasal epithelial cells, which then epithelial cells. This research not only enhances our understanding of the composition and transcriptional signature of porcine nasal mucosal cells but also offers a theoretical foundation for developing alternative models for human respiratory diseases.

**Supplementary Information:**

The online version contains supplementary material available at 10.1186/s13567-024-01403-w.

## Introduction

Recurrent outbreaks of respiratory infectious diseases have had a significant economic impact on the global swine industry [1]. Given that the nasal cavity serves as a primary entry point for pathogens, enhancing nasal mucosal barrier function is critically important for the effective prevention of respiratory infectious diseases [2]. A comprehensive comprehension of the fundamental organization and cellular composition of the nasal mucosa is essential for developing effective strategies to augment the functionality of the nasal mucosal barrier. However, owing to the limitations of available techniques, the study of the nasal cavity has traditionally relied on histomorphology analysis, resulting in a dearth of comprehensive knowledge on the specific cellular composition and interactions within the nasal cavity [3–5]. Single-cell RNA sequencing represents a groundbreaking tool for examining heterogeneous cell populations [6] and has been employed to construct single-cell transcriptome atlases of significant tissues in humans and diverse mammals [7, 8]. Although single-cell atlases of more than 20 porcine tissues have been constructed [9], single-cell atlases from the porcine nasal cavity have yet to be reported.

Owing to their genetic, anatomical, physiological, and immunological similarities with humans, domestic pigs are recognized as critical biomedical models for the study of human diseases [10–13]. They have been extensively utilized in research on various conditions, such as atherosclerosis [14], diabetes [15], and heart disease [16], and have even facilitated the development of porcine kidneys for human xenotransplantation [17]. Multiple studies have revealed that human respiratory viruses, including influenza A virus (IAV) and severe acute respiratory syndrome coronavirus (SARS-CoV), exhibit similar infectious and pathogenic characteristics in pigs [18, 19]. Recently, numerous studies have successfully utilized pigs or cultured porcine respiratory tract tissues to elucidate the infection dynamics and pathogenic mechanisms of various human respiratory viruses [20, 21], which suggests that the porcine nasal cavity is an effective alternative model for human respiratory disease research. Thus, exploring the characteristics of porcine and human nasal mucosa at the cellular and molecular levels warrants further investigation.

In this study, we constructed a single-cell atlas of porcine nasal mucosa and performed a comparative analysis with human single-cell data. We investigated the composition, differentiation trajectories, and intercellular communication of nasal mucosal cells in both pigs and humans. Moreover, we analysed the transcriptional characteristics of cell‒cell junction molecules, pattern recognition receptors (PRRs), and various respiratory virus receptors. This research not only reveals the characteristics of porcine nasal mucosa cells but also offers new insights into the development of alternative porcine nasal models for human respiratory diseases.

## Materials and methods

### Animals

Conventional Duroc × (Landrace × Yorkshire) neonatal piglets (5 days old) were obtained from the Jiangsu Academy of Agricultural Science. All the piglets were seronegative for antibodies against porcine epidemic diarrhea virus, porcine reproductive and respiratory syndrome, transmissible gastroenteritis virus, influenza A virus and porcine circovirus type 2. The animal studies were approved by the Institutional Animal Care and Use Committee of Nanjing Agricultural University and followed the National Institutes of Health guidelines for the performance of animal experiments.

### Cell isolation and single-cell sequencing

Single-cell capture was performed using a Chromium Controller instrument (10× Genomics) as previously described [22]. The mucosa of the nasal respiratory region was dissected from four 5-day-old piglets (two porcine nasal mucosae were mixed into one sample) [5] and then cut into small pieces, which were digested with collagenase type IV (4 mg/mL, Miltenyi Biotec) and hyaluronidase (0.25 mg/mL, Miltenyi Biotec) for 30 min at 37 °C. The released nasal cells were filtered through a 70 μm cell strainer, centrifuged and resuspended in PBS. The activity of the suspended cells was determined by trypan blue staining, and the live cell concentration was adjusted to 1000–2000 cells per microliter. The cells were then captured with a 10× Genomics Chromium Single Cell Instrument after binding to barcoded gel beads. The raw scRNA-seq data were obtained after reverse transcription and RNA sequencing. These processes were completed by Gene Denovo (Guangzhou, China).

### Single-cell RNA sequencing data processing and clustering analysis

The raw sequencing data were processed by using 10X Genomics Cell Ranger software (version 3.1.0, USA), and the sequence reads were aligned to the porcine reference genome Sscrofa 11.1. Single-cell analysis was performed using the Seurat v3 [23]. Cells with fewer than 200 or more than 6500 detected genes were excluded. Cells with unusually high numbers of unique molecular identifiers (UMIs) (≥ 50 000) or mitochondrial gene percentages (≥ 15%) were excluded. Moreover, gel bead-in-emulsion (GEM) mixtures carrying multiple cells were also filtered out. After high-quality cells were retained, we employed the log normalization method to normalize gene expression. To minimize the effects of behavioral conditions and batch variability on clustering, we used Harmony [24] to cluster the data in which the cells were grouped by cell type. Then, principal component analysis (PCA) was used to scale and dimensionally reduce the resulting integrated expression matrix. We used Seurat, which implements a graph-based clustering approach, to cluster the cells. For visualization of clusters, t-distributed stochastic neighbor embedding (t-SNE) was generated using the same PC. Finally, we loaded the log-normalized matrices on the SingleR package for cell type annotation.

### Single-cell RNA sequencing data integration

To integrate our data with published human nasal single-cell RNA sequencing data (including nasal biopsies from three adults and nasal brushings from four adults) [7], we adopted the R package Seurat, and integration was performed as previously reported [25]. The Ensembl genome browser (Ensembl-release 106) was used to convert human (GRCh38) gene names to the corresponding pig gene names before integration. For this analysis, only genes with one-to-one orthologues were utilized, ensuring high quality and consistency. Low-quality cells and genes were excluded from the dataset. Each dataset underwent independent normalization before identifying the features with the highest variability. A standard integration workflow was subsequently employed. Initially, the SelectIntegrationFeatures function was used to identify genes exhibiting consistent variability. Following this, the FindIntegrationAnchors function identified a set of anchors between the human and porcine datasets by leveraging the top 30 dimensions from the canonical correlation analysis to define the neighbor search space. This process facilitated the generation of an integrated dataset using the IntegrateData function. Post-integration, cell cycle effects were regressed out, and clustering analysis was conducted via a series of functions: RunPCA, FindNeighbors, FindClusters, and RunUMAP. To identify differentially expressed genes (DEGs) conserved across datasets, the FindConservedMarkers function was employed. Further analysis was performed to pinpoint species-specific DEGs within selected clusters. An additional column was added to the Seurat object to categorize each cluster by species origin. Relevant clusters were then examined for DEGs using the FindMarkers function. Finally, genes displaying differential expression due to dataset-specific effects or those detected in only one species were excluded from the analysis.

### Marker selection for common cell types

Markers for each of the common cell types were obtained by comparing a certain cell type with all other cell types using the binomial likelihood test embedded in the R package Seurat. In addition, we selected genes that have been published as cell-specific markers.

### Differential expression analysis and gene ontology enrichment analysis

The Wilcoxon rank sum test was used to compare the expression value of each gene in a given cluster against that of the remaining genes to identify significantly upregulated genes. Genes with an adjusted p value less than 0.05 were considered differentially expressed genes (DEGs). We subsequently used the R package clusterProfiler and the annotation R package org.Hs.eg.db to perform Gene Ontology analysis.

### Trajectory analysis

In this study, monocle was used for trajectory construction and pseudotemporal analysis [26]. The gene expression matrix generated by 10x Genomics was imported into Monocle to construct cell differentiation trajectories and visualize cell trajectories for different clusters. Monocle can find genes that are differentially expressed between different clusters and assess the statistical significance of those changes. We identified key genes related to the development and differentiation process with FDR < 1e-5 and grouped genes with similar trends in expression.

### Cell communication

To investigate potential interactions across nasal cell types, CellPhoneDB was used to infer that cell‒cell communication is mediated by ligand‒receptor interactions. Among them, only receptors and ligands expressed in more than a user-specified threshold percentage of the cells in the specific cluster were considered for the analysis (default is 10%). To identify biological relevance, we further used CellPhoneDB software to perform pairwise comparisons between common cell types and analysed the number of significantly enriched ligand‒receptor interactions between two cell types. We defined a *P* value less than 0.05 as significant.

### Cell type distribution of gene expression

The ggplot function of the ggplot2 package in R was used to display the expression of respiratory virus receptors, cell–cell junction genes, and pattern recognition receptors in all epithelial cell types.

### Histological analysis

The specific method used was described previously [27]. In brief, all the animals were euthanized by intravenous injection of pentobarbital sodium (100 mg/kg). The entire nasal region of each animal was subsequently removed, and the skin and muscles around the nose were peeled off. The nasal tissue was fixed in 4% paraformaldehyde stationary solution for 48 h. After fixation, five cross-sectional blocks were selected according to the nasal anatomy of pigs [4] and dehydrated with a graded alcohol series (75%, 85%, 95%, 100%, 100% ethanol). The dehydrated blocks were then embedded in paraffin, serially sliced into 5-µm-thick sections, and mounted on slides. The slices were dried overnight at 37 °C. A cross section of the nasal respiratory region was stained with haematoxylin‒eosin (HE). Integral images were scanned via a BX51 Digital Camera System (Olympus Inc., Tokyo, Japan).

### Immunofluorescence staining and confocal microscopy

To show the distribution of ciliated cells, basal cells and goblet cells in porcine nasal tissue, tissue sections were rinsed and subjected to antigen repair. After being washed with PBS, the sections were treated with 3% hydrogen peroxide solution and incubated at room temperature in the dark for 15 min. Then, the sections were blocked with 5% bovine serum albumin for one hour at 37 ℃ and incubated with rabbit KRT5 antibody. The sections were subsequently incubated with anti-rabbit HRP secondary antibodies at room temperature for 1 h and then with FITC-TSA. For antigen repair, the sections were labelled with an anti-mouse Alexa Fluor 549 secondary antibody (alpha-Tubulin (acetyl K40)) and an anti-mouse Alexa Fluor 633 secondary antibody (MUC5AC) in sequence according to the above steps. The nuclei were stained by incubation with diamidino-2-phenylindole for 10 min and observed under a confocal laser microscope (LSM-710; Zeiss, Oberkochen, Germany).

## Results

### Single-cell transcriptional landscape of porcine nasal mucosal cells

**Figure 1:**
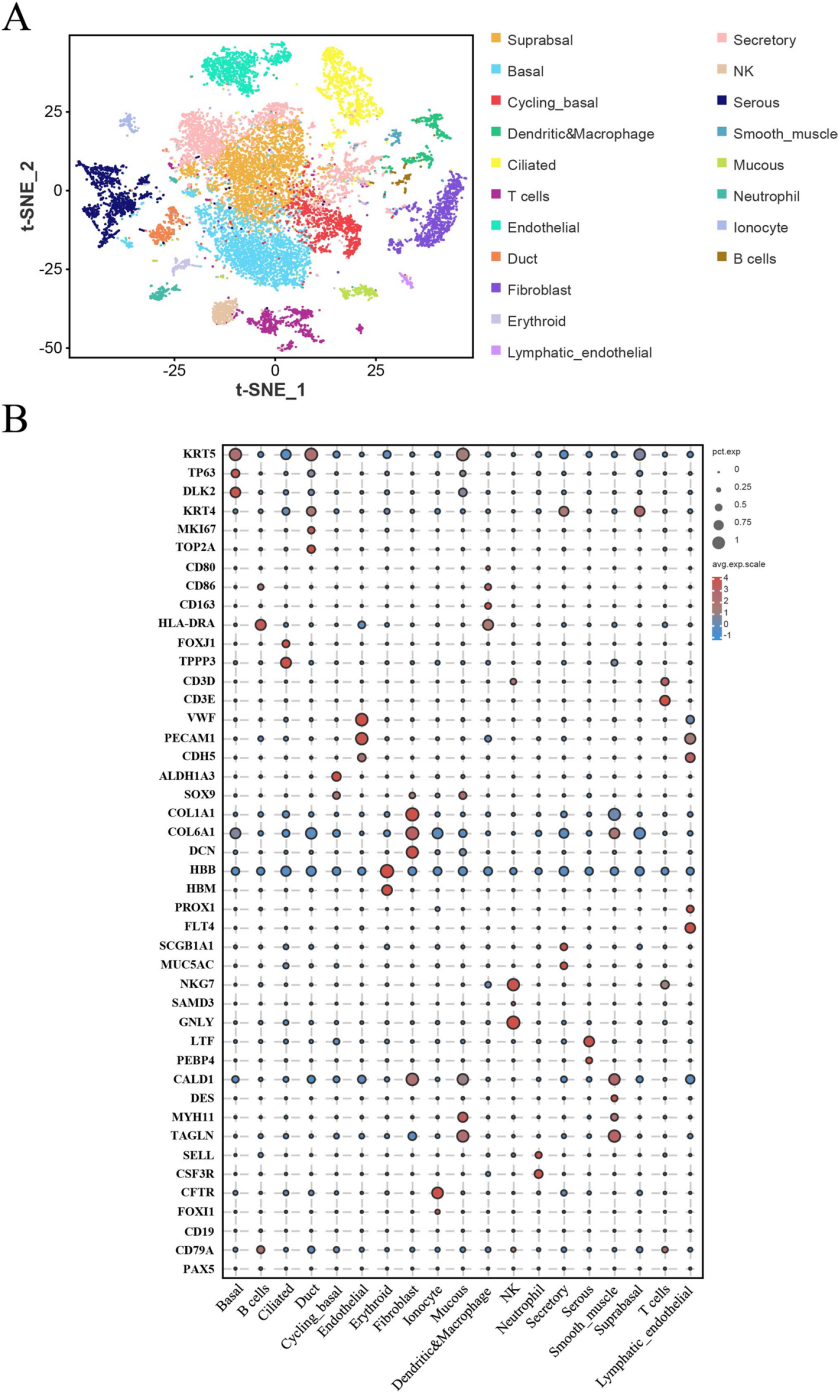
**Single-cell atlas of the porcine nasal mucosa.** **A** t-Stochastic neighbor embedding (tSNE) plots displaying 17 201 cells from porcine nasal mucosa identified as 19 distinct cell types. **B** Dot plots showing marker genes for nasal cell types, with the fraction of expressing cells and average expression within each cell type indicated by dot size and color, respectively.

To analyse the cellular composition of porcine nasal tissue in detail, nasal mucosa samples were collected and dissociated into single-cell suspensions for scRNA-seq. After quality control (including the number of genes, number of mRNA, proportion of mitochondrial gene expression, and multiple filtering), a total of 17 201 cells were obtained (Additional files 1, 2). By tSNE mapping and unsupervised density clustering, these cells were identified as 19 distinct cell types, a classification informed by the differential expression of cell-specific marker genes, as depicted in Figures 1A and B.

First, nine types of epithelial cells were identified on the basis of the expression of cell-specific marker genes. Basal cells, characterized by high expression of KRT5, TP63, and DLK2 [7], serve as tissue-specific stem cells of the airway epithelium. Suprabasal cells exhibit low levels of KRT5 and TP63, along with increasing gradients of KRT4 expression [7, 28]. Cycling basal cells were observed with elevated expression of MKI67 and TOP2A [7]. Secretory cells (including club cells and goblet cells) are found in the nasal mucosa and specifically express SCGB1A1 and MUC5AC [7]. Ciliated cells specifically express FOXJ1 and TPPP3 [7]. Notably, a rare cell type, the ionocyte type, is characterized by high levels of CFTR and FOXI1 expression [29]. Furthermore, three distinct cell types are related to submucosal glands: serous cells (which express high levels of LTF and PEBP4 [7]), mucous cells (which express high levels of MYH11 and TAGLN [30]), and duct cells (which express high levels of ALDH1A3 and SOX9 [31, 32]). Next, five types of stromal cells were identified on the basis of the expression of cell-specific marker genes. These include fibroblasts (COL1A1, COL6A1, and DCN [7, 33]), endothelial cells (VWF, PECAM1, and CDH5 [9, 33]), lymphatic endothelial cells (PROX1 and FLT4 [34, 35]), smooth muscle cells (CALD1 and DES [7, 36]), and erythroid cells (HBM and HBB [37]).

In addition to these cells, various immune cell types were identified according to the expression of cell-specific marker genes. In brief, dendritic cells and macrophages exhibit increased expression of CD80, CD86, CD163 and HLA-DRA [38–40]. Natural killer cells exhibit high expression levels of NKG7, SAMD3, and GNLY [40–42], whereas T cells are enriched in CD3D and CD3E [42]. Neutrophils display high expression of SELL and CSF3R [43], and B cells are identified by the enrichment of CD19, CD79A, and PAX5 [7, 44].

### Distribution characteristics of porcine nasal epithelial cells

**Figure 2:**
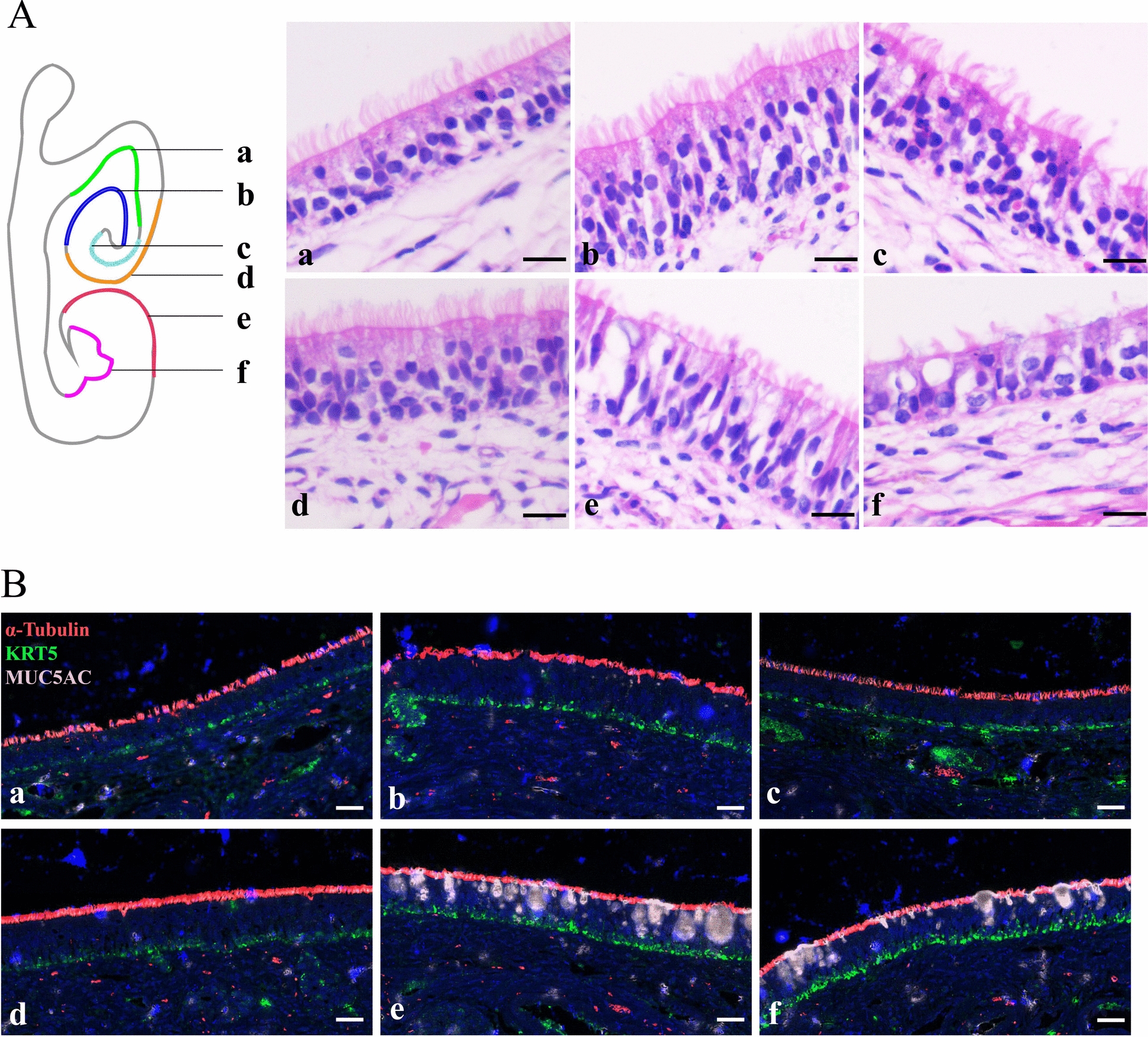
**Morphological characteristics and basal**,** ciliated**,** and goblet cell distributions of porcine nasal mucosa.** **A** The nasal mucosa was separated into six distinct areas (a-f). HE staining of different areas of the pig nasal concha. Scale bars: 10 μm. **B** Immunofluorescence staining for KRT5 (green, representing basal cells), acetylated α-tubulin (red, representing ciliated cells), and MUC5AC (pink, representing goblet cells) in the pig nasal concha. Nuclei are shown in blue (DAPI). Scale bars: 20 μm.

The results of scRNA-seq revealed that basal cells, goblet cells, and ciliated cells are the three main subtypes of epithelial cells. Therefore, we focused on the distribution characteristics of these epithelial cells in porcine nasal mucosa. HE staining revealed notable variations in the morphology and structure of the porcine nasal mucosa at different anatomical sites, resulting in the subsequent division of the nasal mucosa into six distinct regions (a-f) (Figure 2A). The upper regions (a-d) of the nasal mucosa were entirely covered by pseudostratified columnar ciliated epithelia, whose cilia were long and dense. However, the cilia located in the lower regions (e-f) of the nasal mucosa were short and sparse. Moreover, the epithelial layers in regions a and f were relatively thin and consisted of approximately three layers of cells, whereas the epithelial layers in regions b, c, d and e were relatively thin and consisted of approximately five layers of cells (Figure 2A). Immunofluorescence results revealed that acetylated α-tubulin + ciliated cells were widely distributed across the epithelial surface. KRT5 + basal cells were evenly distributed at the base of the epithelium. Interestingly, MUC5AC + goblet cells were exclusively identified in the lower regions of the nasal mucosa and ranged in size and shape (Figure 2B).

### Differences in the composition of porcine and human nasal epithelial cells

**Figure 3:**
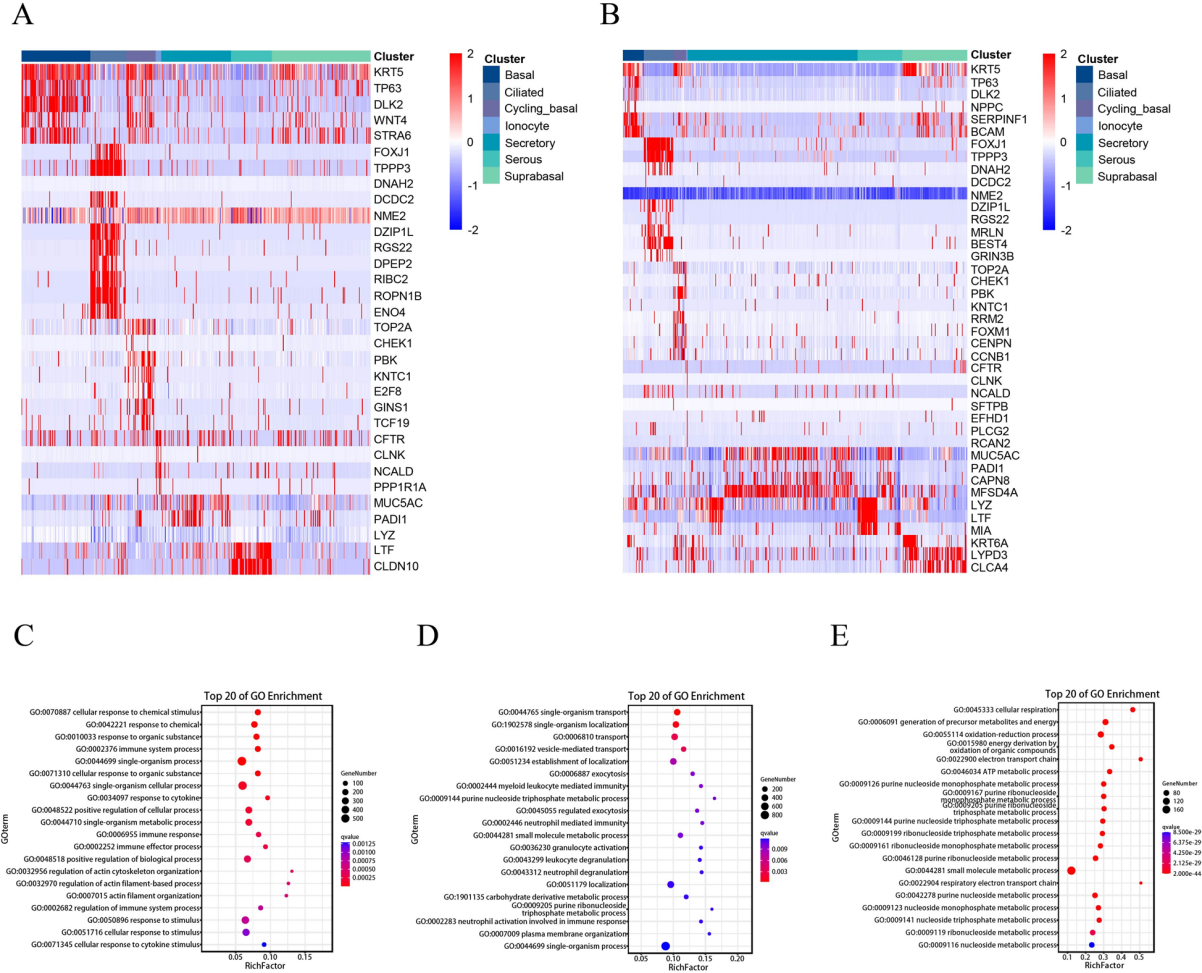
**Differences in the markers and functions of porcine and human nasal epithelial cells.** **A**, **B** Heatmap of marker gene expression in the 7 common cell types for pigs and humans. Representative markers are listed. **A** Pig. **B** Human. **C**, **D** Bubble plot showing the GO pathways in porcine and human secretory cells. The 20 pathways associated with the most significantly enriched biological processes are shown. **C** Pig. **D** Human. **E** Bubble plot showing the GO pathways in porcine duct cells. The 20 pathways associated with the most significantly enriched biological processes are shown.

The physiological similarities between pigs and humans are close enough to make pigs promising xenotransplant donors. However, there is still a lack of relevant research on whether there is consistency in the composition and function of nasal mucosal cells between pigs and humans. Therefore, a comparative analysis of epithelial cell populations was conducted by integrating our own data with accessible single-cell RNA sequencing data from humans. Our findings revealed that eight distinct types of epithelial cells, including basal cells, suprabasal cells, cycling basal cells, secretory cells (goblet cells and club cells), ciliated cells, serous cells, mucous cells and ionocytes, are conserved between porcine and human nasal mucosa. Notably, deuterosomal cells, which are believed to be progenitors of ciliated cells, exist only in the human nasal mucosa. Duct cells were specifically observed in the porcine nasal mucosa. The differential expression of epithelial marker genes in porcine and human nasal mucosa was subsequently analysed. The results revealed high similarity in marker gene expression between porcine and human nasal epithelial cells, such as KRT5 for basal cells and FOXJ1 for ciliated cells. In addition, we screened several unique molecular markers that are expressed in porcine epithelial cells, such as BEST4 for human ciliated cells and DPEP2 for porcine ciliated cells (Figures 3A and B).

To determine whether the function of nasal epithelial cells is conserved between pigs and humans, a Gene Ontology (GO) enrichment analysis of biological processes was conducted. The identical cell types in both pigs and humans presented very similar patterns of enriched pathways (data not shown), with the exception of secretory cells. In porcine nasal secretory cells, the enriched GO terms were predominantly associated with biological reactions, such as response to chemical, response to cytokine, immune response, and response to stimulus (Figure 3C). However, the enriched GO terms in human nasal secretory cells were related primarily to material transport processes, including transport, vesicle-mediated transport, exocytosis, and regulated exocytosis (Figure 3D). Moreover, we focused on the biological functions of porcine duct cells and found that the enriched GO terms in porcine duct cells were always associated with energy metabolism, such as cellular respiration and ATP metabolic processes (Figure 3E).

In terms of immune cells, T cells, dendritic cells, and macrophages are present in both porcine and human nasal mucosa. However, porcine nasal mucosa contains a greater diversity of immune cell types, including NK cells, neutrophils, and B cells.

### Pseudotime analysis of porcine and human nasal epithelial cell development

**Figure 4:**
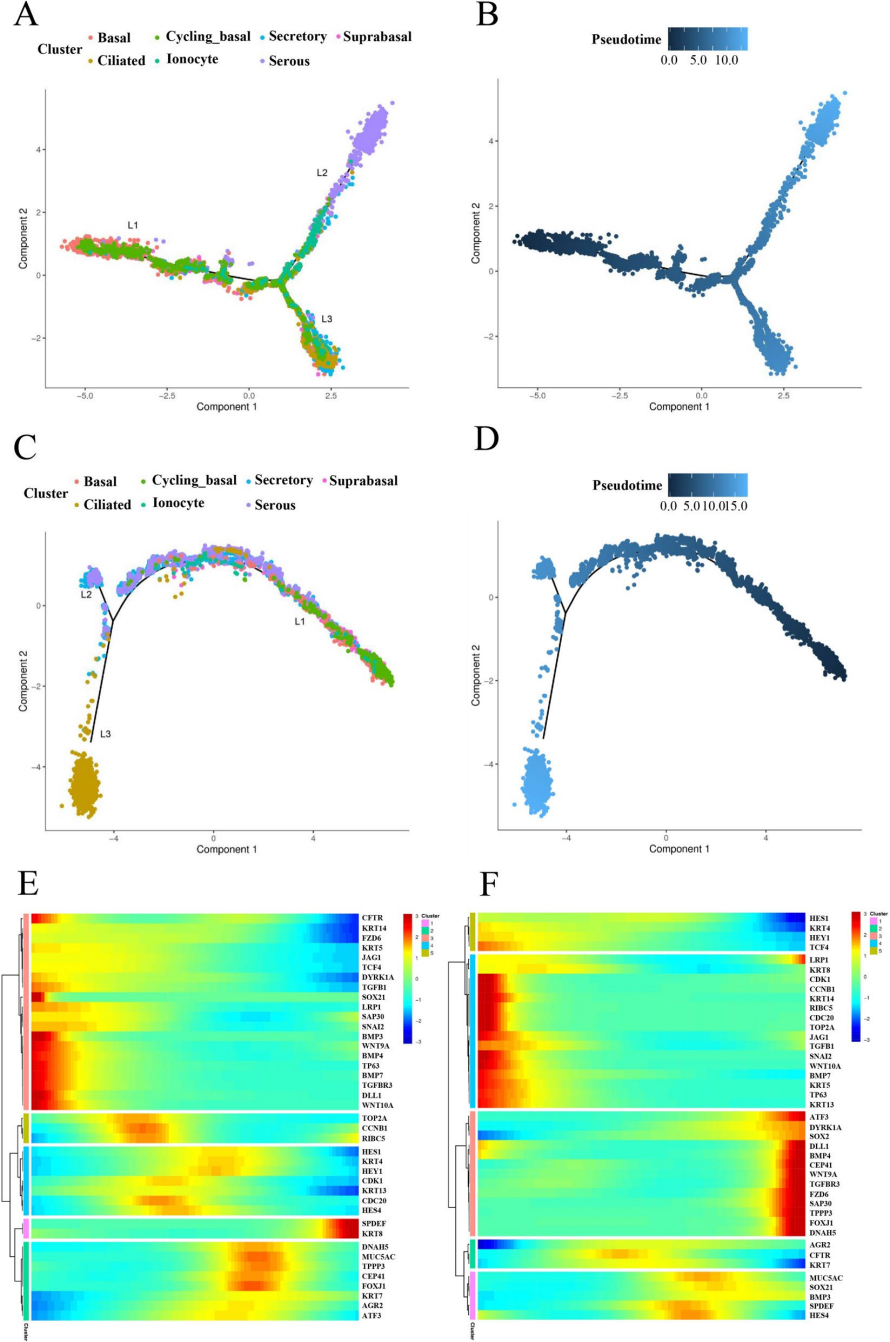
**Pseudotime analysis of porcine and human nasal epithelial cell development.** **A**, **B** Differentiation trajectory of porcine nasal epithelial cells, with each point coloured according to epithelial cell type (**A**) and pseudotime (**B**). **C**, **D** Differentiation trajectory of human nasal epithelial cells, with each point coloured according to the epithelial cell type (**C**) and pseudotime (**D**). **E**, **F** The gene expression levels along the pseudotime trajectories are shown in a heatmap, and representative genes are listed. (**E**) Pig. (**F**) Human.

To investigate the differences in the differentiation process of nasal epithelial cells between pigs and humans, the Monocle tool was employed to reconstruct differentiation trajectories via the pseudotemporal ordering of single cells. The differentiation trajectories of porcine and human nasal epithelial cells exhibited the same trend. Specifically, basal cells, which then differentiate into club cells and further differentiate into ciliated cells or goblet cells, are used as a starting point for nasal epithelial cell differentiation (Figures 4A–D).

A thorough analysis of the dynamics of transcription factors during the development of nasal epithelial cells was subsequently performed. In both pigs and humans, genes associated with the differentiation and development of epithelial cells, such as JAG1, TGFB1, BMP7 and WNT10A, as well as those involved in cell adhesion, such as SNAI2, presented significant expression levels in the early stages. In contrast, certain genes associated with epithelial cell differentiation and development presented increased expression in the later stages in pigs but were more prominent in the early stages in humans (Figures 4E and F). These findings indicate a degree of conservation in the transcription factors responsible for regulating nasal epithelial cell differentiation in both pigs and humans.

### Cell-cell communication between porcine and human nasal epithelial cells

**Figure 5:**
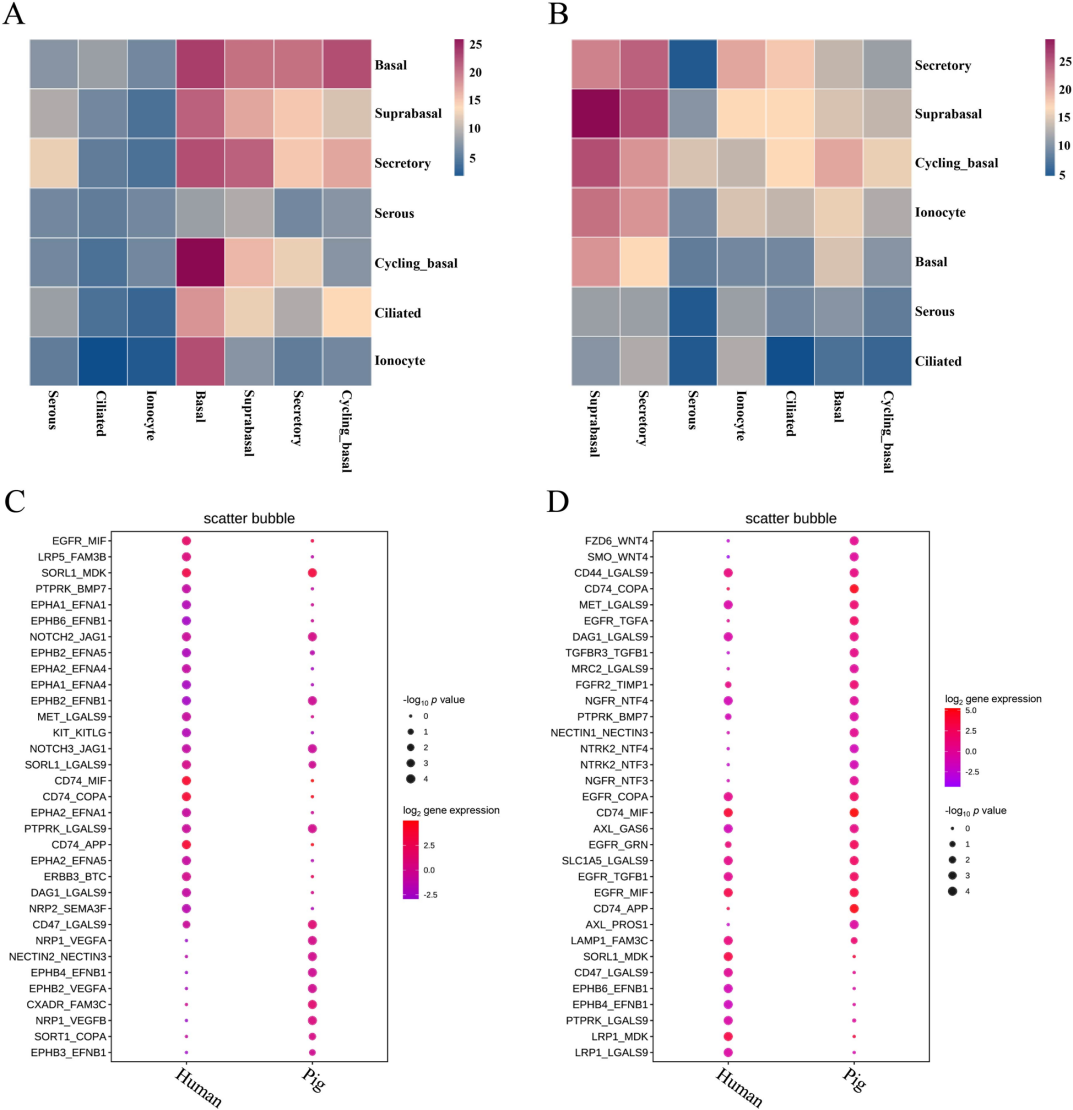
**Cell‒cell communication in nasal epithelial cells between pigs and humans.** **A**, **B** Number of predicted interactions (*P* ≤ 0.05) between nasal epithelial cells based on CellPhoneDB in pigs (**A**) and humans (**B**). **C** Predicted interactions between basal cells and cycling basal cells, comparing humans and pigs. **D** Predicted interactions between suprabasal cells and secretory cells, comparing humans and pigs.

CellPhoneDB was used to explore the cell‒cell interactions between nasal epithelial cells and identify significantly enriched ligand‒receptor pairs. This analysis revealed a greater level of ligand‒receptor interaction in porcine nasal epithelial cells than in human nasal epithelial cells (Figures 5A and B). Specific interactions between basal cells and cycling basal cells almost invariably involve processes related to proliferation and differentiation, such as EGFR-TGFB1, EGFR-MIF, and DAG1-LGALS9 in pigs and humans; FZD6-WNT, TGFBR3-TGFB1, and NGFR-NTF3 in pigs; and EPHB4-EPNB1 and LRP1-MDK in humans. In addition, we identified interactions related to the maintenance of epithelial integrity (NECTIN1-NECTIN3) in pigs (Figure 5C). Similarly, the interactions between suprabasal cells and secretory cells are significantly correlated with proliferation and differentiation. Specifically, NOTCH2-JAG1 and EPHB2-EPNB1 were enriched in both pigs and humans, whereas EPGB4-EPNB1 was enriched exclusively in pigs, and CD74-COPA and EGFR-MIF were enriched exclusively in humans (Figure 5D). These findings suggest that the regulation of nasal epithelial cell development in both pigs and humans involves a combination of conserved and species-specific interactions.

### Transcription characteristics of cell‒cell junction molecules and pattern recognition receptors in porcine and human nasal epithelial cells

**Figure 6:**
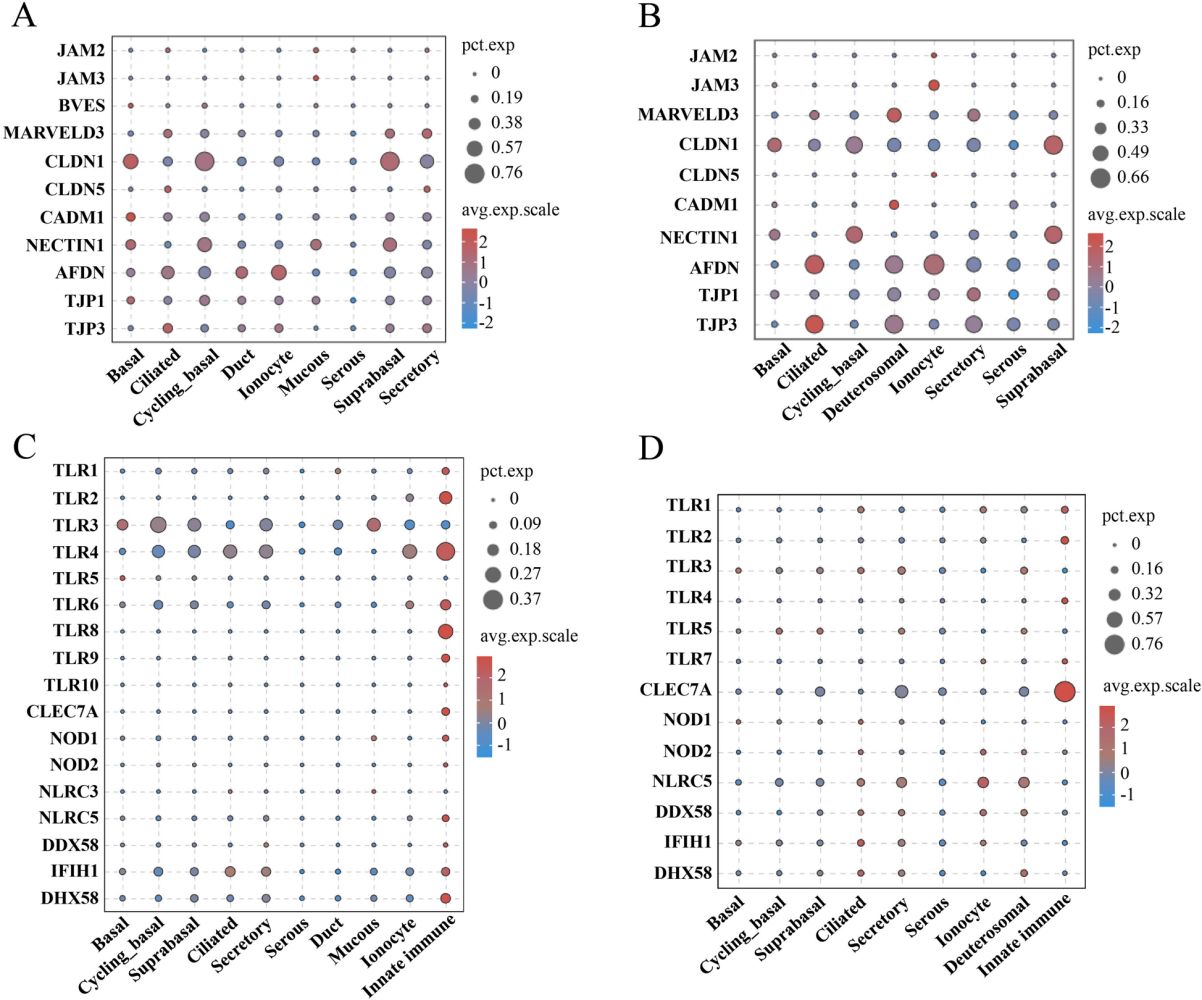
**Expression patterns of cell‒cell junction genes and pattern recognition receptors in porcine and human nasal epithelial cells.** **A**, **B** Bubble plot showing the cell‒cell junction gene expression patterns of epithelial cell types in porcine (**A**) and human (**B**) nasal tissue. **C**, **D** Bubble plot showing the pattern-recognition receptor expression patterns of epithelial cell types and innate immune cells (dendritic cells and macrophages) in porcine (**C**) and human (**D**) nasal regions. The size represents the percentage of cells, and the colour indicates the average scaled expression level.

Cell–cell junctions and pattern recognition receptors (PRRSs) are important parts of the innate immune system. This study examined the expression patterns of genes associated with cell‒cell junction molecules and pattern-recognition receptors in nasal epithelial cells of both pigs and humans. A significant proportion of genes related to cell‒cell junctions presented similar expression profiles across these two species. Specifically, JAM2, JAM3, and CLDN5 presented minimal expression levels in the majority of nasal epithelial cell types. Conversely, CLDN1, NECTIN1, AFDN, TJP1, and TJP3 were highly expressed in most nasal epithelial cell types of both pigs and humans (Figures 6A and B).

However, the expression patterns of PRRS significantly differ between porcine and human nasal epithelial cells. Our analysis revealed that the mRNA transcripts of TLR6, TLR8, TLR9, TLR10, and NLRC3, which are present at low levels in porcine nasal epithelial cells, were absent in human nasal epithelial cells (Figures 6C and D). Additionally, TLR3 and TLR4 exhibited high mRNA levels across various porcine epithelial cell types (Figure 6C), whereas CLEC7A and NLRC5 presented high expression in most human epithelial cell types (Figure 6D). By comparing the expression levels of PRRs in nasal epithelial and innate immune cells (dendritic cells and macrophages), we found that almost all PRRs (except TLR3) had higher expression levels in porcine nasal innate immune cells than in epithelial cells (Figure 6C). In human nasal epithelial cells, TLR1, TLR2, TLR4, TLR7, and CLEC7A presented relatively high expression levels, whereas TLR3, NLRC5, DDX58, IFIH1, and DHX58 were highly expressed in human nasal innate immune cells (Figure 6D).

### Transcriptional characteristics of respiratory virus receptors in porcine and human nasal epithelial cells

**Figure 7:**
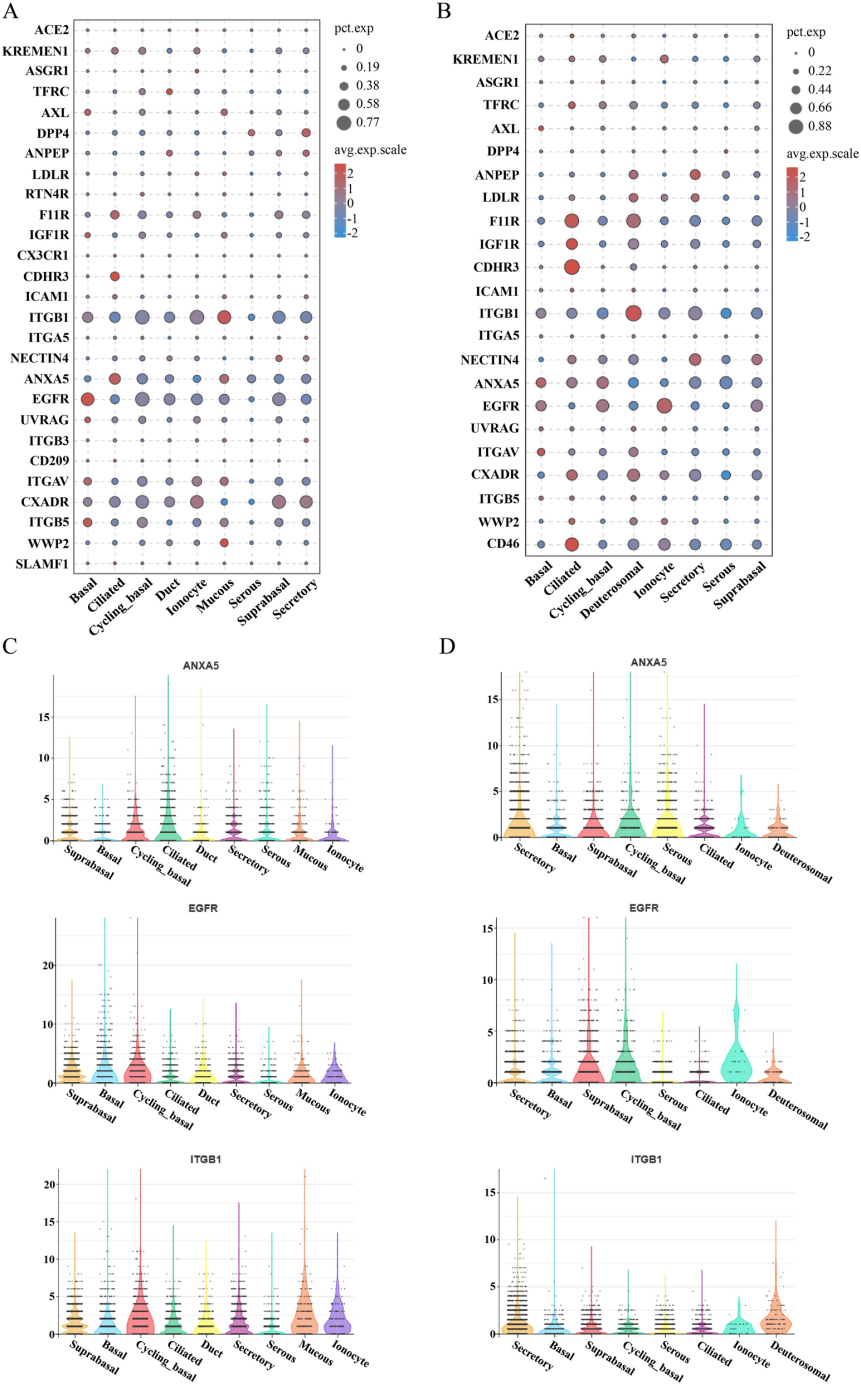
**Expression patterns of respiratory virus receptors in porcine and human nasal epithelial cells.** **A**, **B** Bubble plot showing the virus receptor expression patterns of epithelial cell types in pig (**A**) and human (**B**) nasal regions. The size represents the percentage of cells, and the colour indicates the average scaled expression level. **C**, **D** Violin plots showing the expression of ANXA5, EGFR, and ITGB1 in porcine nasal (**C**) and human nasal (**D**) samples.

The nasal cavity serves as a primary site of entry for respiratory viruses, making it an important target for infection. In this study, the distribution of the expression of multiple respiratory virus receptors in porcine and human nasal epithelial cells was examined. Our findings indicated that most respiratory virus receptors display similar expression profiles across different cell types in both porcine and human nasal epithelial cells (Figure 7A and B, Additional file 4). Specifically, receptors such as ANXA5, EGFR, and ITGB1 were widely expressed across various nasal epithelial cell types in both species (Figure 7C and D). ITGA5 and ASGR1 exhibited significantly low expression levels in all nasal epithelial cell types of both species (Additional file 3). In addition, species-specific differences in the expression of certain virus receptors were detected. Specifically, ANPEP and LDLR were found to have low expression levels in all porcine nasal epithelial cell types but were prominently detected in most human nasal epithelial cell types, particularly secretory cells (Additional file 3). DPP4 was found to be present in a limited number of human nasal epithelial cell types, whereas it was notably abundant in the majority of epithelial cell types in pigs. ACE2 exhibited extremely low mRNA expression in human nasal epithelial cells and was virtually absent in porcine nasal epithelial cells (Additional file 3). Notably, CD46 was expressed solely in human nasal epithelial cells, whereas RTN4R, CX3CR1, ITGB3, CD209, and SLAMF1 were uniquely expressed in porcine nasal epithelial cells (Figures 7A and B). Moreover, ciliated cells presented the highest abundance of viral receptor expression compared with other types of nasal epithelial cells (Figure 7A and B).

## Discussion

Exploring the cellular composition and intercellular interactions of the respiratory mucosa is essential for understanding its defense mechanisms and developing targeted therapeutic strategies [45]. In this study, we performed scRNA-seq on porcine nasal mucosa and generated a single-cell atlas comprising 17 201 cells, which were identified as 19 distinct cell types, including nine epithelial cell types, five stromal cell types, and five immune cell types. The distribution characteristics of the three most representative epithelial cells in the porcine nasal mucosa were subsequently depicted. Our analysis revealed that basal cells and ciliated cells are evenly distributed across the entire mucosal epithelial layer. Ciliated cells, which mediate the mucociliary clearance process, play a critical role in expelling particles and pathogens [46]. Basal cells function as tissue-specific stem cells in the airway, are crucial for long-term self-renewal and are vital for maintaining and repairing the respiratory epithelium [47, 48]. The strategic distribution of ciliated cells and basal cells likely contributes to reducing pathogen colonization and aids in maintaining homeostasis. In contrast, the distribution of goblet cells showed significant regional specificity, which predominated in the lower but was scarce in the upper regions of the porcine nasal mucosa. Considering that goblet cells are responsible for mucus production and that mucus serves as a key component of the mucosal defense mechanism against microbial invasion [49, 50], this uneven distribution suggests potential increased vulnerability to pathogen infection in upper mucosal regions.

Although domestic pigs are generally believed to share histological similarities with humans [51], existing studies have yet to compare the cellular composition and transcriptional characteristics of porcine and human nasal mucosa effectively. Our study revealed that both porcine and human nasal epithelia consist of basal cells, suprabasal cells, cycling basal cells, club cells, goblet cells, ciliated cells, ionocytes, serous cells and mucous cells. Moreover, the marker genes of these nasal epithelial cells are highly conserved between pigs and humans. These findings suggest that the functional roles of the nasal epithelium are likely similar across these two species. Duct cells, which are commonly found in the pancreas, have also been identified in the porcine nasal cavity and exhibit robust energy metabolism activity. Given their role in the secretion of mucin and fluid/electrolytes, particularly HCO_3_^−^ [52–54], the presence of these cells in the porcine nasal cavity may enhance the functional role of the mucosal barrier, meriting further investigation. There is a notable difference in the composition of immune cells in the nasal mucosa between humans and pigs. Given that nasal immune cells are located primarily in the submucosal lamina propria [5], the observed differences may be attributable to the sampling method employed.

Pseudotime analysis, also known as trajectory inference, is used to infer the differentiation trajectory of cells or the evolution process of cell subtypes [55, 56]. Our pseudotime analysis revealed that the differentiation trajectories of nasal epithelial cells in pigs and humans are remarkably similar; both start with basal cells, progress to club cells, and then branch into either ciliated cells or goblet cells under the regulation of different transcription factors. Signalling pathways such as the Notch, Wnt, and BMP/TGFβ pathways play important roles in nasal epithelium development [57]. Our results revealed that the expression patterns of key transcription factors within these pathways (such as JAG1, TGFB1, BMP7 and WNT10A) are similar in both porcine and human nasal epithelial cells. These findings indicate the conserved development of nasal epithelial cells across pigs and humans.

Interactions between cellular populations are essential for the development of the nasal mucosa and the maintenance of the mucosal barrier [58]. Our study revealed that the ligand-receptor pairs between nasal epithelial cells in pigs and humans, such as EGFR-TGFB1, EGFR-MIF, TGFBR3-TGFB1, NGFR-NTF3, EPHB4-EPHB1 and LRP1-MDK, are related to epithelial development [59, 60]. However, the expression patterns of these receptors and ligands are species specific. Taking the interaction between basal cells and cycling basal cells as an example, in pigs, the TGFBR3-TGFB1 and NGFR-NTF3 signalling pathways were specifically activated, whereas in humans, the EPHB4-EPHB1 and LRP1-MDK pathways were specifically activated. These findings suggest that these pathways may uniquely contribute to the development of nasal epithelial cells in each species. Notably, our research also highlighted the unique activation of the NECTIN1-NECTIN3 signalling pathway in pigs, which is known to play a crucial role in forming, maintaining, and modifying cellular junctions [61]. This specific activation implies its potential involvement in establishing early epithelial barriers in pigs.

The maintenance of the physical barrier of the nasal mucosa requires the involvement of cell–cell junctions, including tight junctions, adherens junctions and desmosomes [62, 63]. The observation of similar expression patterns of these junction molecules in porcine and human nasal epithelial cells suggested that the nasal epithelia of pigs and humans have similar physical barrier functions. Pattern recognition receptors (PRRs), which detect pathogens by recognizing pathogen-associated molecular patterns (PAMPs), play a vital role in host defense mechanisms [64]. Our research revealed that pigs exhibit significantly greater variety and expression levels of pattern recognition receptors than humans do. Notably, TLR3, which targets double-stranded RNA from viruses [65, 66], and TLR4, which are activated by bacterial lipopolysaccharides [67, 68], are highly expressed in porcine nasal epithelial cells. These findings suggest that the porcine nasal mucosa may have a stronger innate immune response to infection. PRRs are involved primarily in innate immunity, and macrophages [69] and dendritic cells [70] are the main innate immune cell types. Our findings indicate that the expression levels of PRRs in porcine nasal epithelial cells are significantly lower than those in innate immune cells. These findings suggest that innate immune cells remain the primary mediators of pathogen recognition and the immune response within the porcine nasal mucosa. However, in humans, certain receptors (such as TLR1, TLR2, TLR4, TLR7, and CLEC7A) exhibit relatively high expression levels in nasal epithelial cells, suggesting that human nasal epithelial cells may have evolved a relatively strong innate immune recognition capability.

Pigs are susceptible to a variety of human respiratory pathogens. We found that multiple human respiratory virus receptors, such as ANXA5, ITGB1, ITGA5 and EGFR, exhibit similar expression patterns in porcine and human nasal epithelial cells. Among these, ANXA5 and EGFR have been identified as coreceptors for the influenza virus [71, 72]. The similar expression patterns of these two receptors in both porcine and human nasal epithelial cells may explain why pigs and humans exhibit comparable susceptibility and pathogenic responses to influenza viruses. Notably, our results revealed that ciliated cells presented the highest levels of viral receptor expression. Since ciliated cells are primary targets for RSV and SARS-CoV-2 [73, 74], they are likely more susceptible to infections by various respiratory viruses. However, two issues need to be noted.

First, protein expression levels are crucial in determining cellular functions; thus, investigating the concordance between protein expression and transcription levels in greater detail is essential. Second, the conservation of receptors can affect ligand‒receptor affinity. For example, ACE2 is a well-known receptor for SARS-CoV-2 in humans. Although pigs possess ACE2, differences in amino acid sequences and protein structures make pigs less susceptible to SARS-CoV-2 infection [75, 76]. Additionally, numerous reports indicate that protein modifications can affect the affinity between viruses and their receptors [77]. Consequently, the conservation of receptors across species significantly impacts the susceptibility of viruses.

In conclusion, our study substantially enhances the understanding of the cellular composition and gene expression profiles of the nasal mucosa of domestic pigs. By conducting a comparative analysis of single-cell data from porcine and human nasal mucosa, we identified significant parallels between porcine and human nasal epithelial cells in terms of their cellular composition, differentiation trajectories, and transcription characteristics of cell‒cell junction molecules and various respiratory virus receptors. These insights provide a strong foundation for the use of porcine nasal cavities as alternative models for studying human respiratory diseases.

## Supplementary Information

**Additional file 1. Quality control of single-cell RNA sequencing. **(A) Number of genes, mRNA count, and proportion of mitochondrial gene expression in single cells of each sample before filtering. (B) Number of genes, mRNA count, and proportion of mitochondrial gene expression in single cells of each sample after filtering. (C) tSNE plot of the multiplet distribution in each sample.

**Additional file 2. Cell data quantities in each sample before and after filtering.**

**Additional file 3. Expression of respiratory virus receptors in porcine and human nasal epithelial cells. **(A) Violin plots showing the expression of ITGA5 and ASGR1 in porcine nasal (left panel) and human nasal (right panel) samples. (B) Violin plots showing the expression of ANPEP and LDLR in porcine nasal (left panel) and human nasal (right panel) samples. (C) Violin plots showing the expression of DPP4 and ACE2 in porcine nasal (left panel) and human nasal (right panel) samples.

**Additional file 4. The relationships between receptors and respiratory viruses.**

## Data Availability

The scRNA-seq data of healthy piglets’ nasal mucosa are available in the GEO database with accession number GSE274334.
