# Supplementary figures and images for: Single-cell analysis of nasal epithelial cell development in domestic pigs
Source: Vet Res. 2024 Oct 30;55:140. doi: 10.1186/s13567-024-01403-w (PMC11523856; doi:10.1186/s13567-024-01403-w)

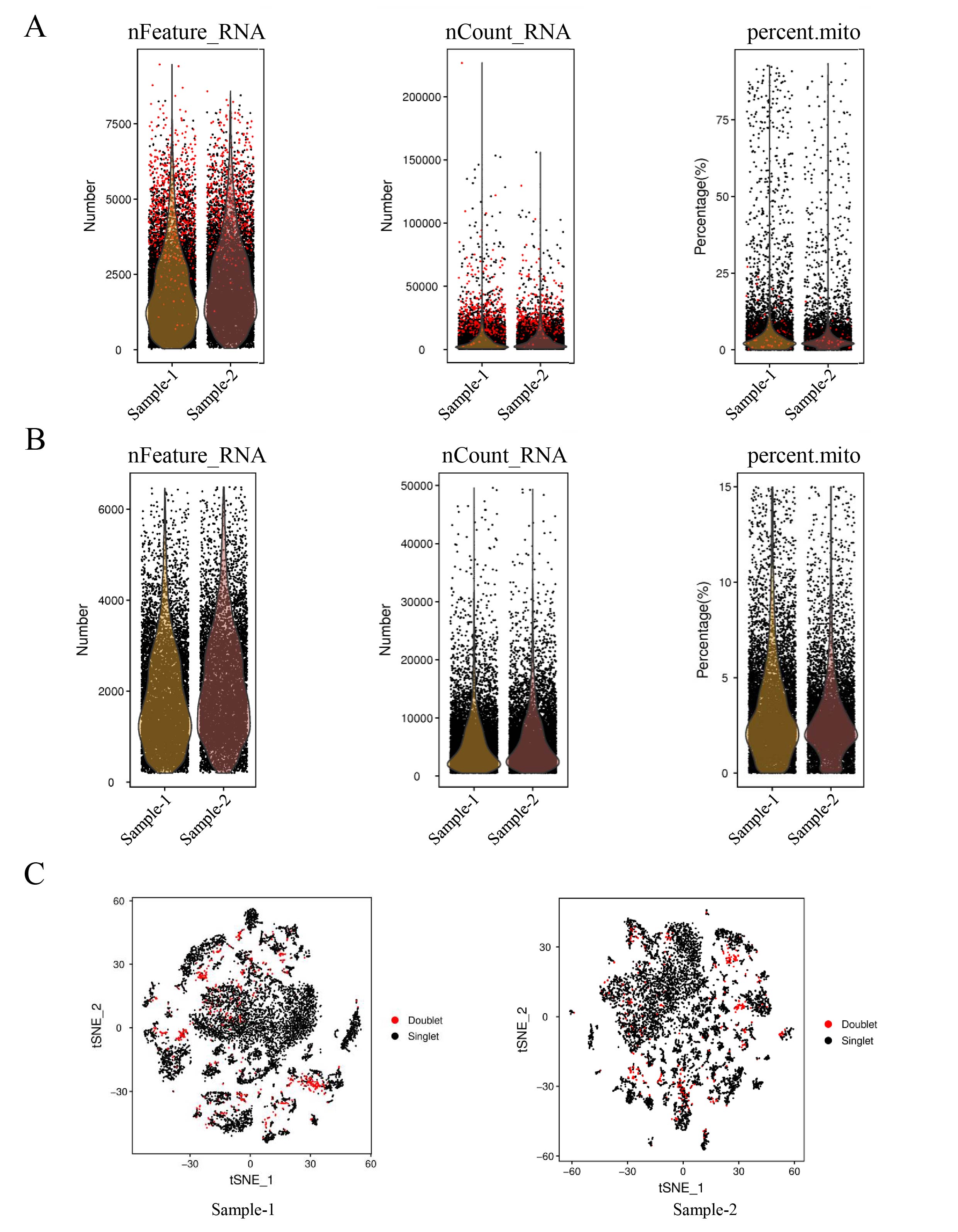

Supplement: Supplementary file 1 — Additional file 1. Quality control of single-cell RNA sequencing. (A) Number of genes, mRNA count, and proportion of mitochondrial gene expression in single cells of each sample before filtering. (B) Number of genes, mRNA count, and proportion of mitochondrial gene expression in single cells of each sample after filtering. (C) tSNE plot of the multiplet distribution in each sample. [file 13567_2024_1403_MOESM1_ESM.jpg]

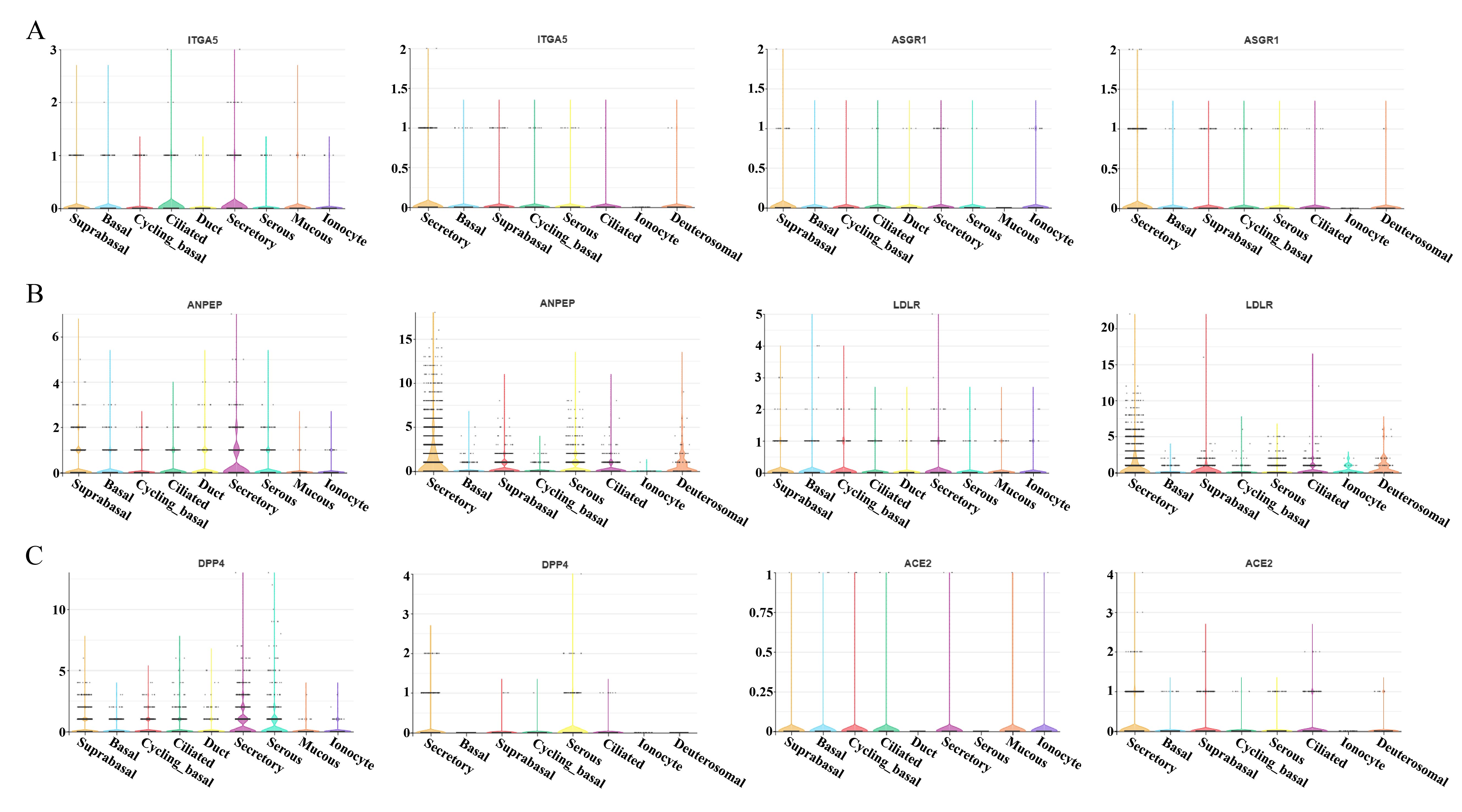

Supplement: Supplementary file 3 — Additional file 3. Expression of respiratory virus receptors in porcine and human nasal epithelial cells. (A) Violin plots showing the expression of ITGA5 and ASGR1 in porcine nasal (left panel) and human nasal (right panel) samples. (B) Violin plots showing the expression of ANPEP and LDLR in porcine nasal (left panel) and human nasal (right panel) samples. (C) Violin plots showing the expression of DPP4 and ACE2 in porcine nasal (left panel) and human nasal (right panel) samples. [file 13567_2024_1403_MOESM3_ESM.jpg]
